# Characterisation of sensor kinase by CD spectroscopy: golden rules and tips

**DOI:** 10.1042/BST20180222

**Published:** 2018-12-04

**Authors:** Giuliano Siligardi, Charlotte S. Hughes, Rohanah Hussain

**Affiliations:** Diamond Light Source Ltd, Harwell Science and Innovation Campus, Didcot, Oxfordshire, U.K.

**Keywords:** circular dichroism, ligand interaction, membrane sensor kinase

## Abstract

This is a review that describes the golden rules and tips on how to characterise the molecular interactions of membrane sensor kinase proteins with ligands using mainly circular dichroism (CD) spectroscopy. CD spectroscopy is essential for this task as any conformational change observed in the far-UV (secondary structures (α-helix, β-strands, poly-proline of type II, β-turns, irregular and folding) and near-UV regions [local environment of the aromatic side-chains of amino acid residues (Phe, Tyr and Trp) and ligands (drugs) and prosthetic groups (porphyrins, cofactors and coenzymes (FMN, FAD, NAD))] upon ligand addition to the protein can be used to determine qualitatively and quantitatively ligand-binding interactions. Advantages of using CD versus other techniques will be discussed. The difference CD spectra of the protein–ligand mixtures calculated subtracting the spectra of the ligand at various molar ratios can be used to determine the type of conformational changes induced by the ligand in terms of the estimated content of the various elements of protein secondary structure. The highly collimated microbeam and high photon flux of Diamond Light Source B23 beamline for synchrotron radiation circular dichroism (SRCD) enable the use of minimal amount of membrane proteins (7.5 µg for a 0.5 mg/ml solution) for high-throughput screening. Several examples of CD titrations of membrane proteins with a variety of ligands are described herein including the protocol tips that would guide the choice of the appropriate parameters to conduct these titrations by CD/SRCD in the best possible way.

## Introduction

### Membrane sensor kinase

Two-component systems (TCSs) are important signalling systems present mostly in prokaryotic organisms and also some lower eukaryotic organisms including the archae [1,2]. Common in both Gram-positive (17 in *Enterococcus faecalis* [3,4]; 34 in *Bacillus subtilis* [5]; 16 in *Staphylococcus aureus* [6,7]) and Gram-negative organisms (30 *Escherichia coli* [8]; *Salmonella enterica*; 63 *Pseudomonas aeruginosa* [9]). TCSs are important mechanisms which enable organisms to respond to the environment and aid their survival.

**Figure 1.**
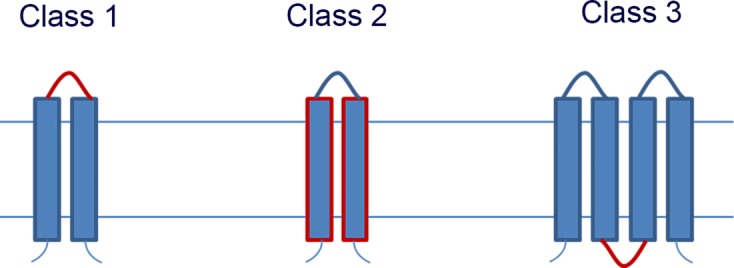
Schematic representation of the classes of histidine kinase receptors based on the location of the ligand sensor domain highlighted in red. The sensor domain can be located extracellularly (Class 1), membrane-embedded and part of the membrane domain itself (Class 2) or cytoplasmically (Class 3). Redrawn from ref. [11].

Comprised of a signal-sensing membrane sensor histidine kinase and a partner soluble response regulator which commonly have DNA-binding and -regulating properties upon phosphorylation, TCSs regulate a variety of responses to environmental changes. Three classes of a membrane sensor kinases exist and are characterised by the location of the ligand-binding domain (LBD) [2,10,11]; the LBDs of Class 1 are located extracellularly, Class 2 are membrane-embedded, and Class 3 have cytosolic LBDs (Figure 1). Of the three classes, Class 1 is the most common.

Membrane proteins are more complex to express and purify in sufficient quantities due to low expression fidelity, low yields (mg quantities for medium-scale grow-ups) and challenging solubilisation and purification protocols that require detergents [12]. Furthermore, identifying ideal crystallisation conditions for the protein of interest, once sufficient quantities of pure protein have been obtained, is a major challenge for obtaining high-resolution structural information regarding the protein in the absence and presence of ligands. It is for these reasons that, to date, there is only one crystal structure for full-length sensor histidine kinase in the Protein Data Bank (PBD) [13]. Obtaining high-resolution structural information for a membrane protein of interest is technically challenging and experiments are often relatively long term. The lipid cubic-phase approach of crystallisation has proved particularly successful for the crystallisation of membrane proteins [14–16] and has been applied to domains of histidine sensor kinases for the determination of structural changes occurring as a result of ligand binding [17]. Furthermore, cryogenic electron microscopy (cryo-EM) has also been shown to be particularly useful with regard to high-resolution structure determination of membrane proteins [18–23]; therefore, it is anticipated that future experimental strategies will adopt cryo-EM as an additional tool for the determination of high-resolution structures of sensor histidine kinases.

Owing to the technical constraints of obtaining high-resolution structural details of membrane proteins, complementary low-resolution and relatively rapid methods can be used to determine the structural activities of a protein occurring during ligand binding. Methods including but not limited to analytical ultracentrifugation (AUC), nuclear magnetic resonance (NMR) spectroscopy, isothermal titration calorimetry (ITC), circular dichroism (CD) and synchrotron radiation circular dichroism (SRCD) can be used to characterise the structural features of a membrane protein of interest in the absence and presence of ligands, the details of each method shall be discussed in this review.

### Pros and cons of the use of AUC, NMR and ITC

**AUC** like CD does not require the need of immobilise, modify or label the protein or the ligand [24]. AUC has been used to characterise the globular form of detergent-solubilised membrane proteins, in particular the oligomerisation species in solution [25–28]. The effect of auto-phosphorylation [29] has been also reported using AUC. Owing to the experimental set-up, titrations are not possible; therefore, AUC used only for qualitative studies. AUC is a powerful method which requires samples meet three criteria for successful measurements: (i) the sample has a distinguishable optical property which can be measured using one of the above optical systems, (ii) sedimentation occurs at a reasonable rate within the experimental set-up and (iii) the material does not interact with the sample cell. Within these constraints, AUC is capable of analysing samples with molecular mass ranges of 100–10^8^ Da ranging from peptides, proteins, DNA to viruses [30].

**NMR** is the spectroscopic technique to obtain protein conformational information in solution at atomic resolution, in particular for the structured element of secondary structure [31–34], can be also used for the investigation of the ligand-binding properties and activities of membrane proteins [35–37]. Solution-state measurements require higher concentrations of protein (20–50 mg) for ideal experimental conditions and significant effort. A limitation is the requirement of ^13^C or ^15^N labelling of samples and the multi-dimensional measurements to achieve the highest spatial resolution that is expensive and time-consuming. Considering the conformation of proteins is affected by the environment that can determine the function/activity, the identification of the more relevant conditions by CD spectroscopy will better focus the effort and maximise the impact of the NMR study. In particular, the screening of the conditions to induce conformational changes from natively disordered to folded proteins is much better achieved by CD first and then by NMR for higher resolution than searching the optimum conditions by solely NMR.

**ITC:** The principle of ITC measurements is that heat is either absorbed or released during binding and these changes are monitored by the calorimeter. Two cells are monitored during an experiment, a reference cell containing water and a sample cell. The calorimeter monitors the temperature of both cells, aiming to keep both at the same temperature. During a titration, small aliquots of the ligand are incrementally added to the host in the sample cell and the changes in temperature are monitored. The total change in heat of the sample is monitored directly as a proportional measure of the amount binding occurring. The energy required to maintain a constant temperature in both cells after each addition of ligand is measured and when plotted against the molar ratio of ligand at each point enables the determination of binding constants (*K*_d_), the stoichiometry (*n*), enthalpy (Δ*H*) and entropy (Δ*S*). There are many advantages in using ITC for ligand-binding studies including no molecular mass restrictions for the binding components and a highly precise, sensitive and reproducible method, which is able to measure a wide range of binding affinities from 10^−2^ to 10^−9^ M and heat changes as small as 0.1 µcal [38,39]. Although ITC does not require labelling and immobilisation of the protein, the technique can still be affected by negative false results when the bound ligand is displacing many bound solvent molecules leading to no net or little heat absorbed or released. Another disadvantage is the complexity of assessing the specific heat contribution in systems containing more than one ligand-binding site [39]. ITC has been used successfully for the ligand-binding studies of membrane proteins [40] including GPCRs [41], ion channels [42], transporters [43], pumps [44] and histidine membrane sensor kinases [45].

Apart from NMR, AUC and ITC do not provide direct information about the types of conformational changes induced by ligand binding and this is one of the main reasons why CD/SRCD should be used to determine directly the conformational behaviour of biologically important molecules as a function of environment such as temperature, pressure, pH, solvent composition, ionic strength, chemical agents, surfactants and ligand-binding interactions.

### CD spectroscopy

Since the first reported heterologous expression and purification of a full-length sensor kinase, PrrB (RegB), over 15 years ago [46], research into the mechanisms of TCSs has included investigating the activity and structural features of TCS proteins. Until now, 16 of the sensory kinases of *E. faecalis* have been heterologously expressed in *E. coli* [47] and purified as full-length and active proteins for use in phosphorylation activity assays [46–49] for spectroscopic investigations into structural effects of ligand binding using Diamond B23 beamline for SRCD [50].

**Figure 2.**
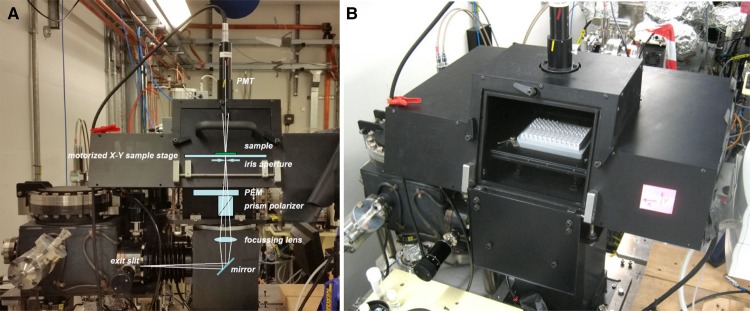
Schematic of the vertical chamber available at B23. (**A**) Diagram of the arrangements of optical elements in the vertical chamber leading to the sample chamber (**B**) housing an X–Y motorised stage which is able to hold a 384- or 96-well plate as shown in (**B**). Image redrawn from Hussain et al. [52]. 1, 0.02 and 0.001 cm fixed pathlength cells are available for 96-well multi-cells, and volume-dependent pathlengths are available to 384-well plate. The pathlength of the wells of the 384-well plate is determined by the volume of sample in the cells, and therefore, accurate pipetting is required by the User.

**Figure 3.**
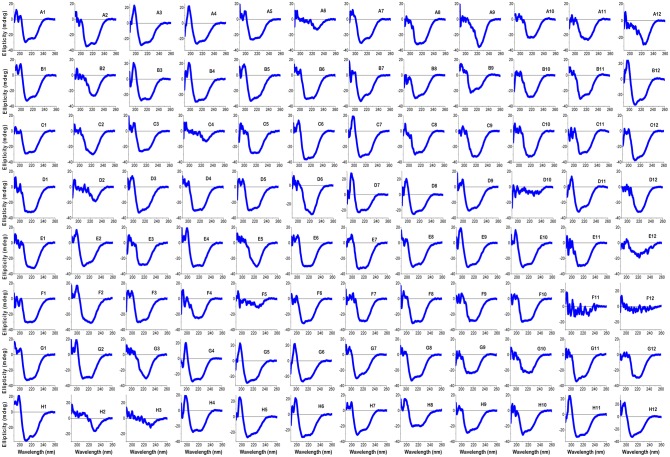

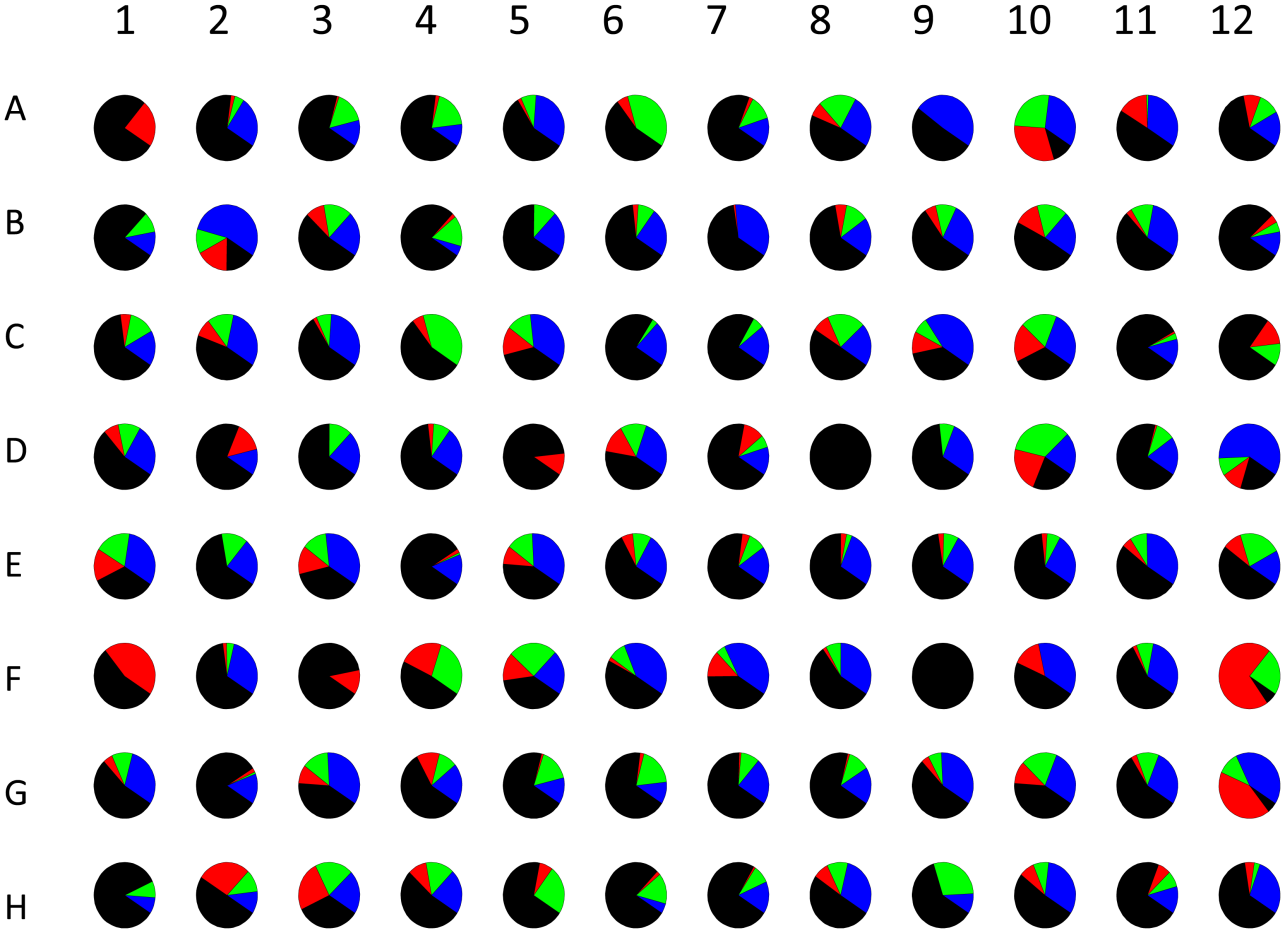
Analysis of HT-SRCD measurements conducted on myoglobin in 96 different buffer conditions. (**A**) Difference spectra for myoglobin (0.1 mg/ml) in 10 mM phosphate buffer were collected in the far-UV region (190–260 nm) by dispensing 60 µl into each 6.6 mm diameter well and subtraction of buffer baselines for each well and (**B**) secondary structure estimations for each condition using the CONTIN algorithm and SP43 database (190–240 nm, estimating alpha-Helix type 1 (H1) and alpha-Helix type 2 (H2) (black), beta-Strand type 1 (S1) and beta-Strand type 2 (S2) (red), Turns (T) (green), Unordered (U) structures (navy)). All analysis and graphical output were obtained using CDApps [53]. Image redrawn from ref. [52].

Membrane proteins such as sensor kinases are usually expressed in small quantities requiring methods that produce high quality data but using the lowest amount of protein. This can be achieved using the Diamond Light Source B23 beamline for SRCD spectroscopy as the beam light of B23 is 10-times brighter than that of Xe arc lamp used in benchtop CD instruments [51] (Figure 2). Most importantly, such an increased brightness is delivered as a highly collimated microbeam with a diameter that can vary from 1 to 0.05 mm compared with 8–3 mm for conventional benchtop CD instruments. This unique feature enables the study of comparatively smaller sample volumes for a variety of microcuvette cells, flat capillaries and the use of multiwell plates (Figure 3) [52].

The use of 96- or 384-well plates for high-throughput CD (HTCD) screening is possible because the beamline station is equipped with a novel and unique vertical sample compartment that holds a horizontal X–Y motorised stage that can accommodate multiplates. The vertical chamber is versatile as it can be used not only to measure HTCD, but the CD of thin films currently imaging the surface at high spatial resolution of 50 µm of areas of several millimetre square. Compared with the time required for measurement including cell cleaning for the same number of samples using individual cuvettes (∼20 min per sample, 32 h for 96 samples), the well plate system (2.5–5 h for a complete measurement and 2 h for cleaning and drying of the multiplate) is much more time-efficient, an advantage for Users of B23 who are granted limited beamtime per visit. The HTCD screening feature is very popular among the B23 users who study the folding of membrane proteins of which crystal structures are still elusive [54–56], the determination of ligand-binding interactions and detergent formulation screening for crystallisation purposes [52].

The determination of a protein–ligand interaction qualitatively is achieved by comparing both far-UV and near-UV regions the spectrum of the observed mixture of protein–ligand at a (1:X) molar ratio with that calculated by adding the spectrum of the protein with that of the ligand at an X molar ratio. In the far-UV region, usually, the CD is dominated by the conformational changes induced to the protein folding by the ligand addition. In this case, the comparison can also be done between the CD spectrum of the protein and the difference spectrum can be calculated by subtracting the CD spectrum of the ligand at a X molar ratio from that of the mixture of protein–ligand at a (1:X) molar ratio. With this approximation, the protein secondary structure content can be estimated using various algorithms for secondary structure estimation from CD/SRCD data such as CONTINLL [57–59], CDSSTR, SELCON3 [58] and BeStSel [60] more appropriate for β-rich proteins and assessed what elements of secondary structure have been affected. The fact that no conformational changes are observed in the far-UV region does not demonstrate the lack of ligand interactions. Interactions may not lead to net changes in secondary structure composition but instead a rearrangement of the protein native elements of secondary structure within the global 3D structure. Such reorganisations, detected and discriminated in the far-UV region (180–250 nm), can lead to the exposure or concealment of portions of the protein from a solvent that can be further probed in the near-UV region (250–330 nm). This is often the case [61–63], but it has to be confirmed by repeating the CD titration in the near-UV region (250–330 nm) where the CD spectral changes induced by the ligand interaction can be the results of several contributions: (i) the perturbation of the local environment within 6 Å of the aromatic side-chains of Phe at ∼260 nm, Tyr at ∼275 nm and Trp at ∼270 and 290 nm; (ii) the changes in dihedral angles of protein disulfide bonds and (iii) the twisting of the skeleton of the ligand molecule viewed by its chromophores absorbing in the near-UV region.

A dedicated suite of programs, called CDApps [53], have been developed at B23 to process data files collected at the beamline or any other CD data uploaded in ascii format. Different analysis options are available within the program (1 to *n* spectra, titration, UV denaturation, thermal melt and 96-well plate).

The 1 to *n* spectra option allows the User to obtain averaged, baseline subtracted and normalised spectra of multiple files in one workbook, provided that the wavelength ranges are the same for sample and baseline. UV denaturation experiments are analysed by subtracting the baseline spectrum from each scan of the file, treating each spectral scan individually. The changes in signal at a specific wavelength can be monitored to determine the kinetics of the change as a function of scan number that if multiplied by the time required for a single scan is also a measure of the irradiation time in the UV-denaturation assay [64].

Thermal melt similarly analyses each scan individually rather than as a function of temperature. The change of CD signal magnitude at a specific wavelength for each temperature can be fitted with a Gibbs–Helmholtz equation [65,66], a derivative of the Boltzmann equation that gives information about the rate of changes and the thermodynamic parameters associated with it.

For files collected using the 96-well multiplate, each of the 96 scans are individually treated as a single spectrum (Figure 3a).

With CDApps, the CD/SRCD data can be then analysed through the program in terms of protein secondary structure content using either or three of publicly available algorithms CONTINLL [57–59], CDSSTR and SECLON3 [58] (Figure 3b).

**Figure 4.**
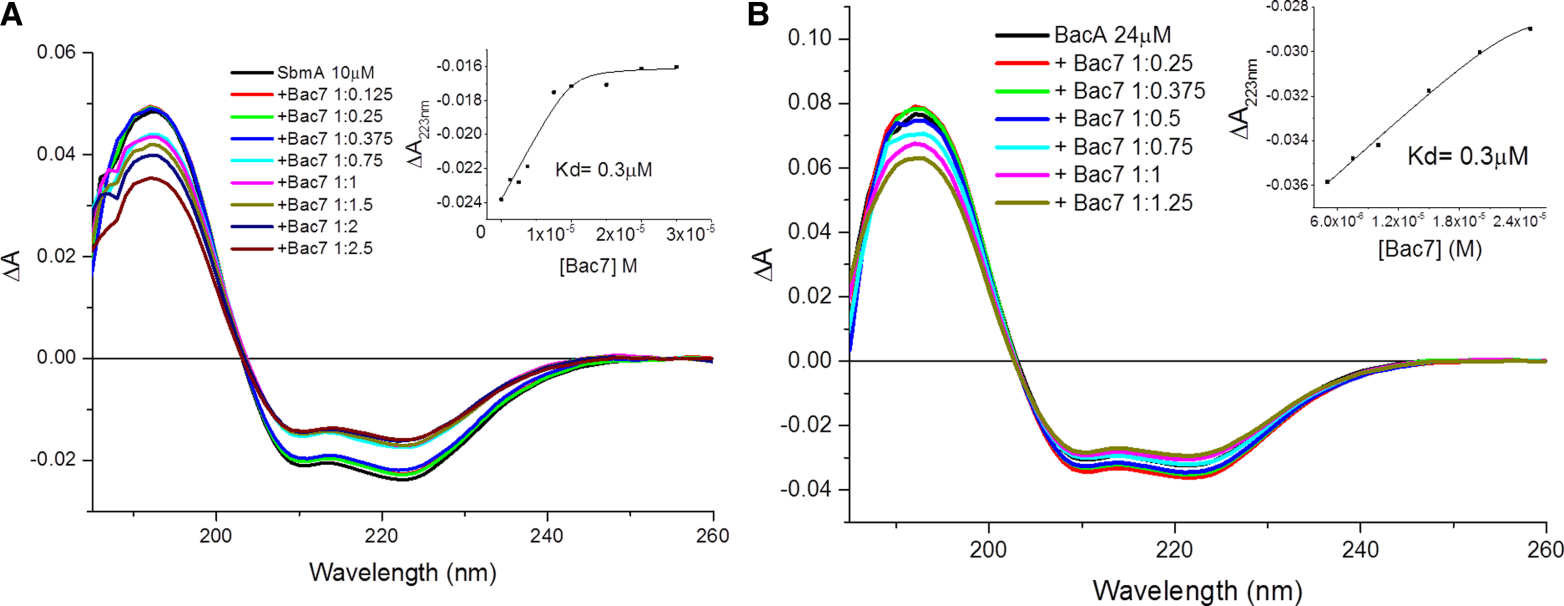
Binding interaction of Bac7 with SbmA and BacA. Titration of Bac7 binding to (**A**) SbmA and (**B**) BacA. Measurements conducted in the far-UV region (185–260 nm). Measurements collected using a 0.02 cm pathlength and (**A**) 10 µM SbmA or (**B**) 24 µM BacA typically prepared in 20 mM Tris–HCl, pH 7.5, and 20 mM NaCl containing 0.03% (w/v) DDM. Both inset graphs show the change in absorbance at 223 nm against concentration of Bac7. Image redrawn from ref. [56].

**Figure 5.**
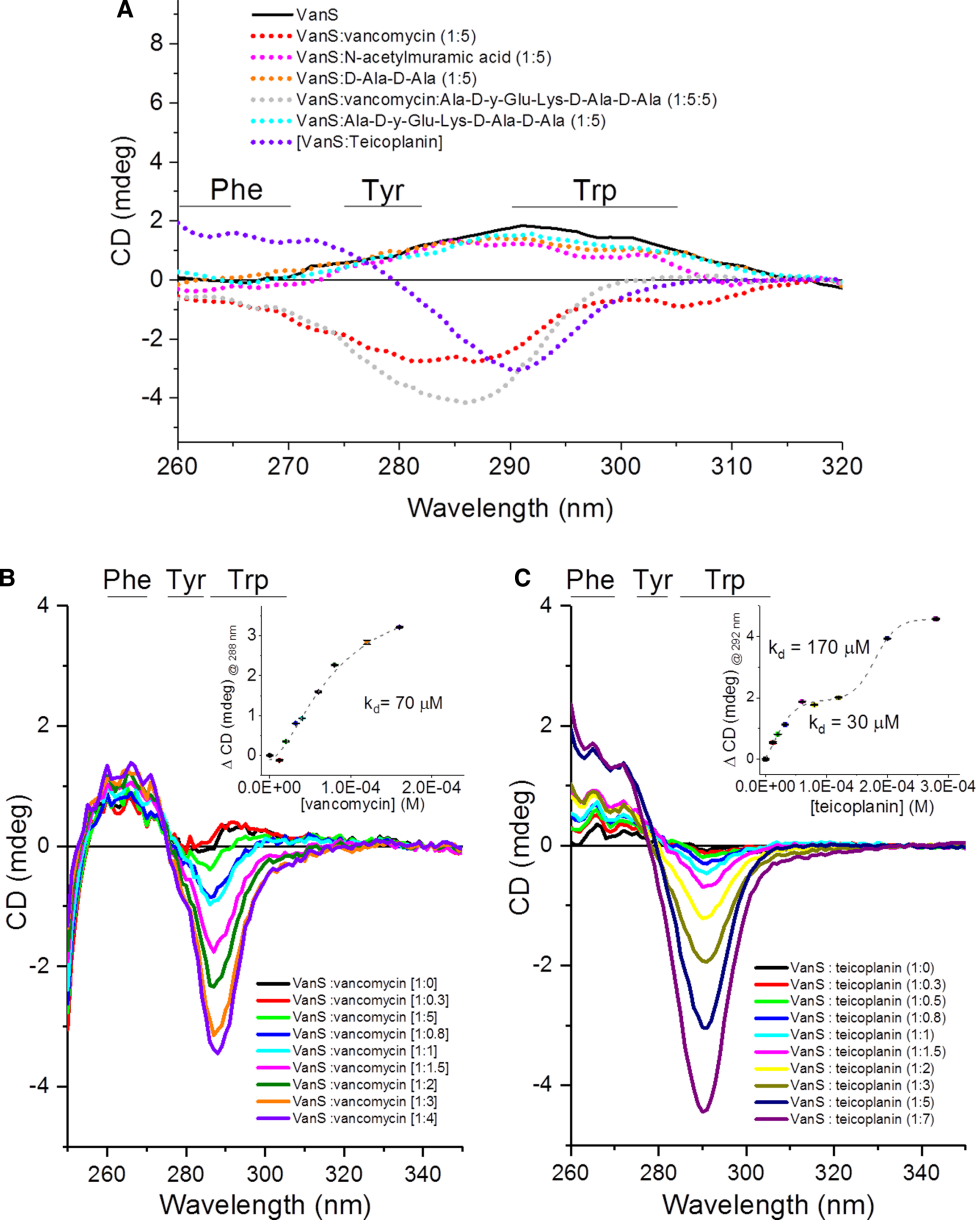
Titration of glycopeptide antibiotic binding to VanS_A_. Measurements were collected in the near-UV region (250–340 nm) employing a 1 cm pathlength to measure 1 mg/ml of VanS_A_ in 10 mM Tris pH 8.0, 10% glycerol and 0.025% (w/v) DDM. Difference spectra are displayed, and analysis performed using CDApps [53]. (**A**) Qualitative binding assay for cell wall component and glycopeptides vancomycin and teicoplanin binding to VanS_A_, (**B**) titration of vancomycin to VanS_A_ and (**C**) titration of teicoplanin to VanS_A_. Image redrawn from ref. [62].

In the last two decades, the determination of the binding dissociation constant *K*_d_ of ligands titrated into proteins by CD spectroscopy has been possible using a non-linear regression analysis method [67,68]. The plotting of the CD intensity of the maximum wavelength of the spectral change versus the mole fraction of the ligand can be used to determine the stoichiometry of the binding interaction in solution and the Hill equation [69] enables the assessment whether the binding is cooperative or not. This analysis can be applied to far-UV sensitive to the protein secondary structure, the backbone folding (Figure 4) and near-UV sensitive to the local environment of the aromatic side-chains of Phe (260 nm), Tyr (275 nm) and Trp (270 and 295 nm) and dihedral angles of disulfide bonds (250 and >310 nm) (Figure 5).

It is important to note that there are many methods for the determination of protein–ligand-binding interactions such as fluorescence, ITC, SPR and AUC. However, none of these techniques can determine directly, unlike CD spectroscopy whether protein conformational changes occurred as a result of ligand-binding interactions. Does a disordered protein or a loop fold upon ligand interaction and of what type: more alpha helical or beta strand? This question is also pertinent for membrane proteins of classes 1 and 3.

**Figure 6.**
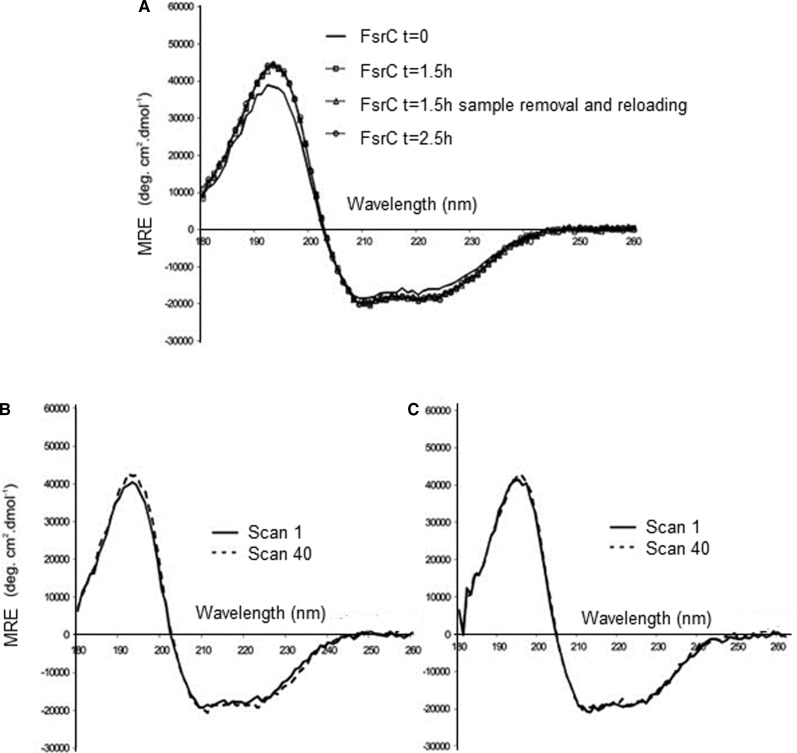
Stability of purified FsrC in buffer containing 0.02% DDM. The stability of FsrC (6 µM) in 10 mM sodium phosphate pH 7.5, 0.02% DDM (w/v) was monitored in the far-UV region (180–260 nm) using 0.02 cm pathlength (**A**) over 2.5 h and after 40 scans alone (**B**) and in the presence of GBAP (**C**). Image redrawn from ref. [50].

The CD titration has been successfully used to assess and quantify the binding property of several membrane proteins such as FsrC [56] (Figure 5), Ace1 [70], SbmA (Figure 4) [69], BacA (Figure 4) [69] and VanS_A_ (Figures 3 and 6) [55,56,62,71,72].

### Rules of thumb

General rules of thumb are reported in Hussain et al. [52], indicating the protein concentration of 0.3–0.5 mg/ml as the optimum concentration to measure a good signal-to-noise ratio CD spectrum using a 0.02 cm cuvette cell pathlength. Following the Beer–Lambert law [*A* = *ε c l*, where *A* is the absorbance (no unit), *ε* the molar absorption coefficient (M^−1^ cm^−1^), *c* the concentration in molarity (M) and *l* the cell pathlength in cm] for measurements of more concentrated proteins, for example 10 folds concentrated, a cuvette cell with a pathlength of 0.002 cm is therefore required to retain the same absorbance and hence the same signal-to-noise ratio spectrum quality. To achieve a good CD/SRCD titration experiment, the recommended absorption of the solution (solvent + solute) to be measured has to be within >0.4–1.6< range. Smaller than 0.4 the spectrum will be too noisy and greater than 1.6 the spectrum will be distorted with modern bespoke CD instruments or extremely noisy for older CD instruments. In the majority of CD instruments [Jasco, Olis, Applied Photophysics Ltd and Biologic], the detector is a photomultiplier tube (PMT) on which a high tension (HT) voltage is applied to amplify the current generated by the transmitted light passing through the sample in the cuvette cells. The detector of the Chirascan Plus or Chirascan V100 is the avalanche photodiode (APD) detector operating at fixed voltage (∼1850 V) and for which the change of gains is made to be equivalent to that of the PMT, the higher the HT, the higher the gain, so that the parameter to optimise the performance of the APD detector can be comparable to that of the PMT.

The HT voltage that recorded simultaneously during CD measurements on benchtop and synchrotron CD instruments is a measure of the amplification of the signal. As different wavelengths have different energies, the HT applied or equivalent for the APD detector will vary to optimise the signal arising from the transmitted light hitting the detector. The transmitted photon flux will differ for strongly absorbing materials at the chromophore's wavelength. At low sample concentration, the applied voltage will be low while at higher sample concentration less transmitted light reached the detector requiring a higher voltage. The golden rule is to follow the CD/SRCD instrument manufacturer recommendations that for the majority of instruments operating in the UV–Visible region is not to exceed the 600–650 V otherwise spectral distortions are generated. In fact with older, pre-millennium, CD instruments, the cut-off due to the lower transmitted light reaching the detector due to higher sample or solvent UV absorption was much less ambiguous than with modern instruments. Measuring the HT of the incident light or using pre-recoded values in the case of Jasco CD instruments, the HT up to 600–650 V can be converted with a good approximation to the UV absorption spectrum that should be simultaneously measured with each CD spectrum. This will enable to confirm that the right concentration range is achieved and that the sample or solvent is devoid of impurities and that for protein–ligand titrations, the concentrations and in particular the molar ratios are appropriate and correct.

Chloride anions absorbs in the far-UV region and hence is a good practice to determine the transparency range of the buffer used to prepare the protein and ligand solutions [63,73]. If a high chloride anion concentration is necessary, a narrower pathlength than 0.02 cm and higher protein concentration are required. For a protein of ∼10 mg/ml concentration in 150 mM NaCl, a 10 µm demountable cuvette will enable the scanning of CD/SRCD spectrum in the 185–250 nm wavelength range without cut-off and spectral distortions that may result in the spectral over analysis and interpretation. If salts are required at high concentrations (100–150 mM), fluoride salts can be an alternative to sodium or potassium chloride salts [61,73] as they are more transparent in the far-UV region. However, the protein folding in fluoride anions does not necessarily remain the same as that in chloride anions and this has to be confirmed experimentally. The challenge will be to use a suitable cell pathlength and protein concentration to compare the widest overlapping spectral region between the protein in chloride and fluoride anion solutions. Sulfate salts are also suitable alternatives [61], for which the same consideration discussed for protein in fluoride anion solutions needs to be made.

The binding of ligands to proteins is conveniently revealed by CD spectroscopy when detectable CD spectral changes are observed. These changes can be induced in the far-UV region by affecting the backbone folding or in the near-UV region by perturbing the local environments of the aromatic side chain chromophores and changing the dihedral angles of present disulfide bonds or both. For these reasons, any CD titration should be carried out in both spectral regions.

A CD titration starts with the spectrum of the protein and subsequent spectra are scanned by adding small aliquots of ligand at incrementally increased molar ratios until the observed change reaches a saturation point (it is important to reach saturation as it will increase the accuracy of the *K*_d_). If the ligand is achiral, the titration reaches saturation when there are no more changes as the excess of free ligand will show no CD. For chiral ligands, when saturation is reached, further addition of free chiral ligand will show an enhanced CD intensity proportional to the increased molar ratio, whereas upon reaching saturation the CD changes are getting smaller. In this case, as both protein and ligand possess CD, the data are reduced to one chiral component by subtracting the spectra of the equivalent CD spectra of each increased concentration of ligand from those of the mixture of protein and ligand at various molar ratios called difference CD spectra. The wavelength that presents the biggest change in magnitude is plotted versus the increased molar concentration of ligand and the data are fitted using a non-linear regression analysis [68]. As with qualitative measurements, the total absorbance should be between 0.4 and 1.5 for the appropriate cell pathlength, and for absorption >1.5, the pathlength have to be reduced accordingly. This can be easily and conveniently done by using cuvette cells with double pathlength like 1 cm in one direction and 0.5 or 0.4 cm in the orthogonal one. Simply rotating the cuvette, the titration can be carried out remaining within the recommended range without having to repeat the titration at different protein concentrations increasing the probability of errors and inaccuracy of the measurements.

Often ligand binding may occur without inducing any CD spectral change in the far-UV region, but nevertheless observable in the near-UV region can be used to detect unambiguously molecular interactions both qualitatively and quantitatively. However, both regions may rarely show any CD changes upon ligand addition for example with transparent ligands, such as short alkyl chain fatty acids and amines [74]. In these cases, thermal denaturation studies have been used for the determination of the effects of ligand binding on the thermal stability of the protein using benchtop CD instruments [75,76] as well as Diamond B23 beamline [55,56,62,71,72,77]. For thermal decay measurements, a single protein sample is subjected to increasing temperatures (of pre-determined increments) and the change in CD signal at a specific wavelength is plotted for each temperature step to determine the melting temperature and the thermodynamic Δ*H* and Δ*S* of the system using the Boltzmann equation.

The photon flux of the light source at the B23 beamline is ∼10-fold more intense than that of the Xe lamp of benchtop CD instruments [51] and can lead to denaturation of a protein of interest. This phenomenon has led the development at B23 of UV-denaturation assay for the study of the photostability of proteins with and without ligands [51,74,78], proving very useful methods to determine the binding interactions of transparent ligands that otherwise very difficult to study with the more traditional titration and thermal methods [74] and for the same reasons for the screening of protein formulations with a variety of buffers and excipients [79]. The protein UV-denaturation assay is now successfully routinely carried out that is part of the B23 facilities available to the user community.

The protein UV-denaturation assay simply consists of scanning repeated consecutive spectra that can vary from 4 to 100 depending on the protein photostability. For a set of proteins, the wild-type and mutants, the plot CD intensity at single wavelength versus the number of scans — representing the average irradiation time that for the far-UV region (185–260 nm) is ∼3 min — can be seen as the rate of protein denaturation and use to quantify the relative protein photostability.

The stability of a protein can be monitored over a period of time prior to any manipulation to ensure that its folding is maintained. This is particularly important for membrane proteins where the solubility in detergents is critical. For the protein FsrC [70], it was observed that the preparation of the solutions with the right concentration from the stock solution for the SRCD titration study required an incubation time at room temperature of ∼1.5 h to equilibrate a stable protein folding for each aliquot addition of the ligand (Figure 6). Without that incubation time that had to be implemented for each spectrum with an increased amount of ligand for the titration, the determination of the *K*_d_ would not have been possible as the spectral variability would have been too big.

Since 2015, it is possible to carry out high-throughput SRCD measurements at B23 using 96-well plates under the similar parameters of pathlength and wavelength range [52]. The data processing and data analysis have been greatly facilitated by the in-house developed CD Apps [53]. The CDApps enables the project to be automated that means only 96-well plate sample preparation and loading is required to start the SRCD measurements, for which the software treats as a single measurement.

**Figure 7.**
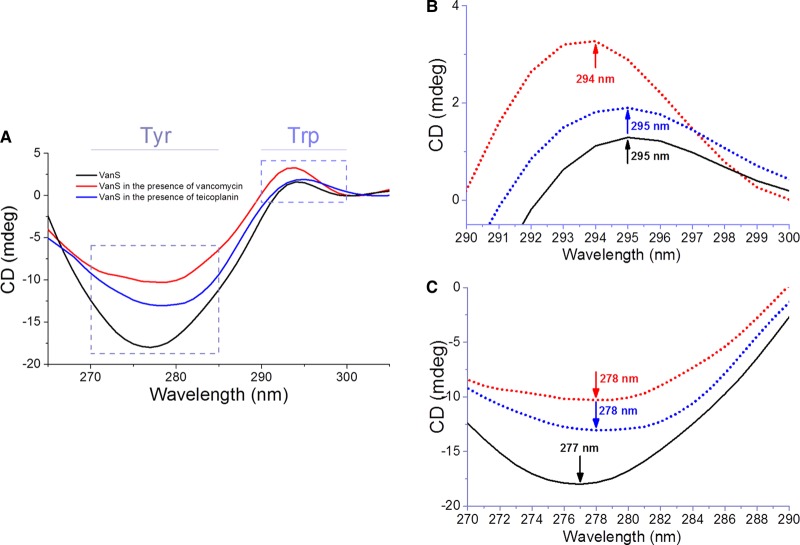
Difference MCD spectrum of VanS_A_ in the presence of glycopeptide antibiotics vancomycin and teicoplanin. (**A**) Qualitative binding of glycopeptide antbiotics to VanS_A_, and magnified graphs of the (**B**) tryptophan region and (**C**) tyrosine region. Data collected using a 1 cm pathlength to monitor VanS_A_ (1 mg/ml) in 10 mM Tris pH 8.0, 10% glycerol and 0.025% (w/v) DDM. Image redrawn from ref. [62].

Modifications to the samples chamber include the addition of a magnetic sample holder. Placing the sample in the magnetic field parallel to the direction of the propagation of beamlight enables the measurement of magnetic circular dichroism (MCD) [80]. In general, a molecule absorbs UV light if it has a chromophore in which an electronic transition can occur which is a displacement of charge: an electron moves from the ground state to the excited state. For chiral molecules, this is a helical displacement of charge originated by the combination of a displacement of charge (electronic transition, for example π–π* transition of an amide bond) and the rotation of charge (magnetic transition, for example the n–π* of the carbonyl group of the amide bond). A non-chiral (achiral) molecules subjected to an external magnetic field coplanar to the direction of the incident beamlight will generate an induced MCD. The MCD generated with one orientation of the magnetic field will cancel the MCD generated with the opposite direction of the magnetic field as the molecule is achiral. For chiral molecule, though the MCD will also have a CD contribution that needs to be subtracted by measuring the CD of the sample without the external magnetic field. Without entering into much detail, MCD can provide information about the electronic state of atoms (in the X-ray region) or molecule (in the UV–Vis region), in particular the oxidation and spin states of transition metals of metalloproteins and haeme proteins. The aromatic side-chains of tryptophan and, to lesser extent, tyrosine amino acid residues of proteins do possess strong intrinsic MCD that is rather sensitive to ligand-binding interactions. Ligands without indole moiety will have no MCD contribution simplifying the task of identifying whether Trp residues are directly involved in the protein-binding site like the example illustrated in Figure 7 of the binding interaction of glycopeptide antibiotics vancomycin and teicoplanin to VanS_A_ membrane protein [62].

### UV–Vis and fluorescence spectroscopy in characterisation of sensor kinase

**Figure 8.**
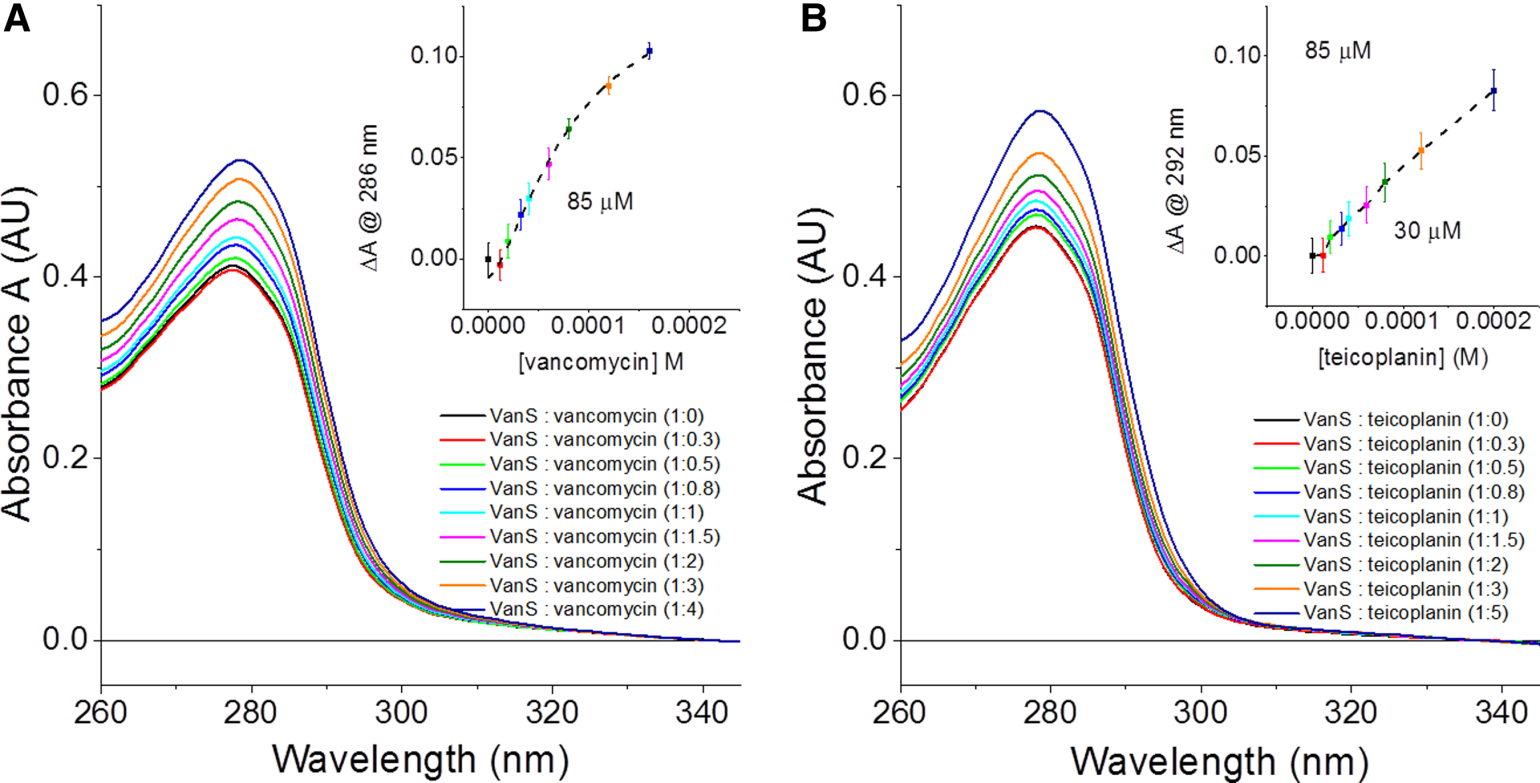
Absorbance spectra of titrations for binding of glycopeptide antibiotics to VanS_A_. (**A**) Absorbance spectra for vancomycin titration and (inset) fitting of difference in absorbance for titration, (**B**) absorbance spectra for teicoplanin titration and (inset) fitting of difference in absorbance for titration. Measurements were collected using a 1 cm pathlength, to monitor 1 mg/ml of VanS_A_ in 10 mM Tris pH 8.0, 10% glycerol and 0.025% (w/v) DDM. Image redrawn from ref. [62].

In the case of titrations of glycopeptide antibiotics vancomycin and teicoplanin binding to VanS_A_, absorbance measurements (Figure 8) collected simultaneously with CD spectra enabled the determination of the dissociation constant *K*_d_ obtained by SRCD.

**Figure 9.**
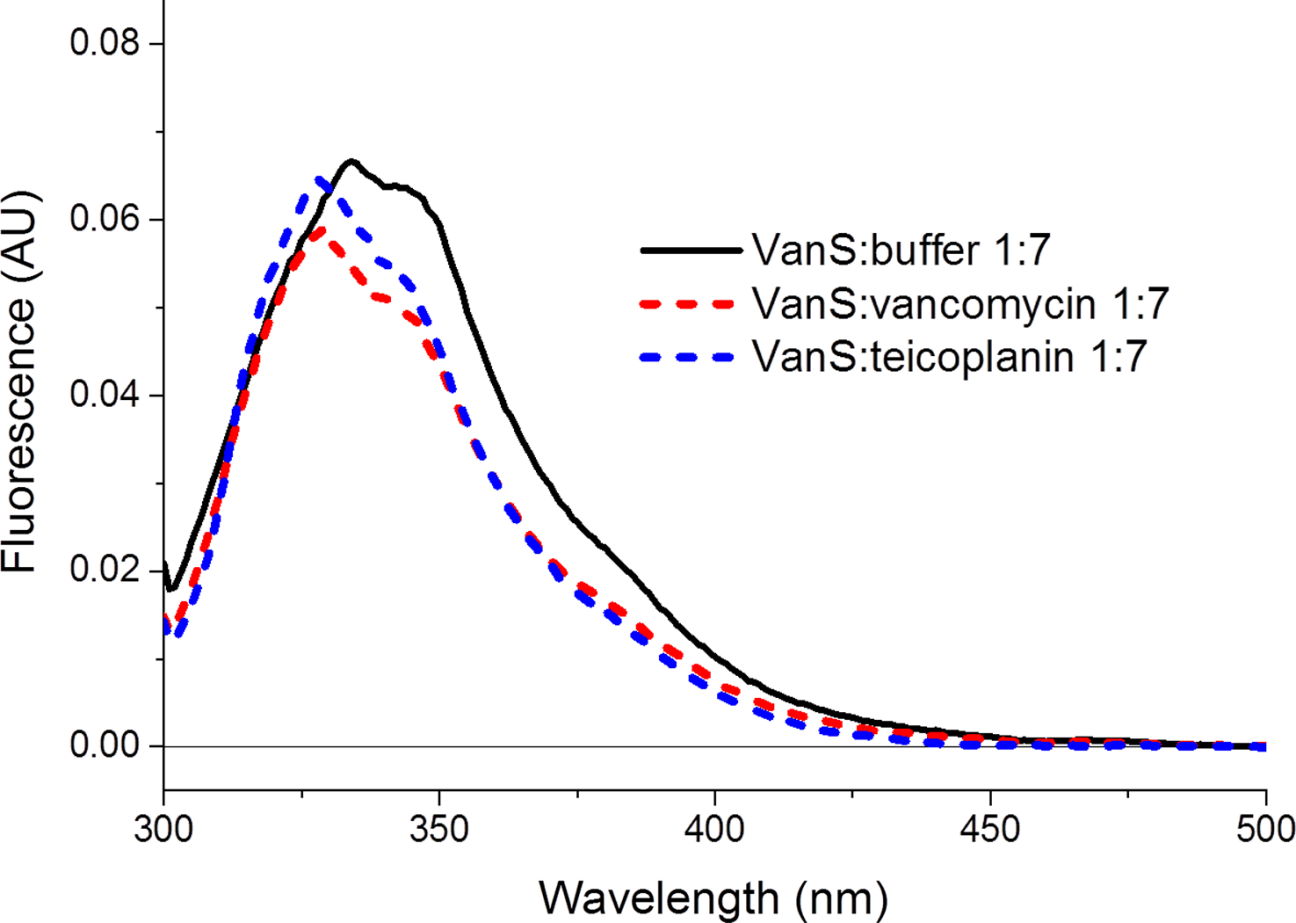
Difference fluorescence spectra for VanS in the presence of glycopeptide antibiotics. VanS_A_ (0.2 mg/ml) in 10 mM Tris pH 8.0, 10% glycerol and 0.025% (w/v) DDM was monitored in the absence and presence of ligands. Using a 1 cm pathlength cell (1 cm × 1 cm, 4 mm × 4 mm window) and excitation wavelength 295 ± 5 nm, emission recorded between 300–500 nm, 1 nm increments. Image redrawn from ref. [62].

Fluorescence spectroscopy is a very sensitive method to determine protein–ligand-binding interactions. A part from the limitation of requiring a suitable fluorophore that if it is not present it has to be chemically conjugated with the possibility of affecting the results as positive or negative results, the major limitation is the inherent inaccuracy of determining the *K*_d_ of the interaction if the ligand absorption overlaps with that of the fluorophore excitation (Figure 9). Often this is ignored and even if corrections are applied, it is still an approximation that needs to be confirmed with other independent methods. Excitation wavelengths need to be within a small range near to the maximum excitation wavelength. Emission should not be recorded overlapping the excitation wavelength range to avoid misinterpretation of results. Similar to CD, high concentrations of fluorophores should be avoided to prevent the ‘inner-filter’ effect on samples whereby too high concentrations lead to a high population within the sample that prevents a sufficient absorption of light by the samples leading insufficient detection of the fluorescence emittance shielded by the crowded environment [81]. Low concentrations, in the region of 0.2 mg/ml using a 1 cm cuvette cell, are regularly employed to prevent the ‘inner effect’ in addition to acknowledging the absorbance contribution of the ligand.

### Main tips on good practice of CD measurements

1) **Optimum protein concentration** to measure the **far-UV** (185–250 nm) CD spectrum. The optimum peptide/protein concentration is 0.3–0.5 mg/ml using 0.02 cm pathlength cuvette. Increasing the sample concentration the cuvette pathlength has to be decreased accordingly following the Beer's Law as described above.
2) **Chloride anion and buffer concentrations** — Depending on these concentrations, the optimum protein concentration and cell pathlength have to be chosen appropriately in order to scan a spectrum in the 185–250 nm without any distortion or cut-off. For NaCl concentration <20 mM, the protein concentration of 0.3–0.5 mg/ml in 0.02 cm pathlength can still be used to measure CD in the far-UV region to characterise the protein folding. With NaCl of 50 mM, the protein concentration should be raised to 1 mg/ml using 0.01 cm pathlength cuvette. With NaCl of 100–150 mM, ∼2 mg/ml of protein concentration using 0.005 cm pathlength cuvette is required. With NaCl >150–250 mM, 5 mg/ml of protein concentration using 0.002 cm and finally NaCl, 500 mM, concentration of protein 10–15 mg/ml should be used using 0.0001 cm pathlength cell cuvette. With these higher NaCl concentrations, attention has to made for wavelength lower than 190 nm as distortion or cut-off might still be occur.

With **phosphate**, **MES** (2-(*N*-morpholino)ethanesulfonic acid) or **CHES** (*N*-cyclohexyl-2-aminoethanesulfonic acid) buffers, the sulfone moiety will absorb in the far-UV region; however, 20 mM routinely used will have minimal impact on the quality of the spectra. Adding NaCl to MES the guidelines above will apply.

**Figure 10.**
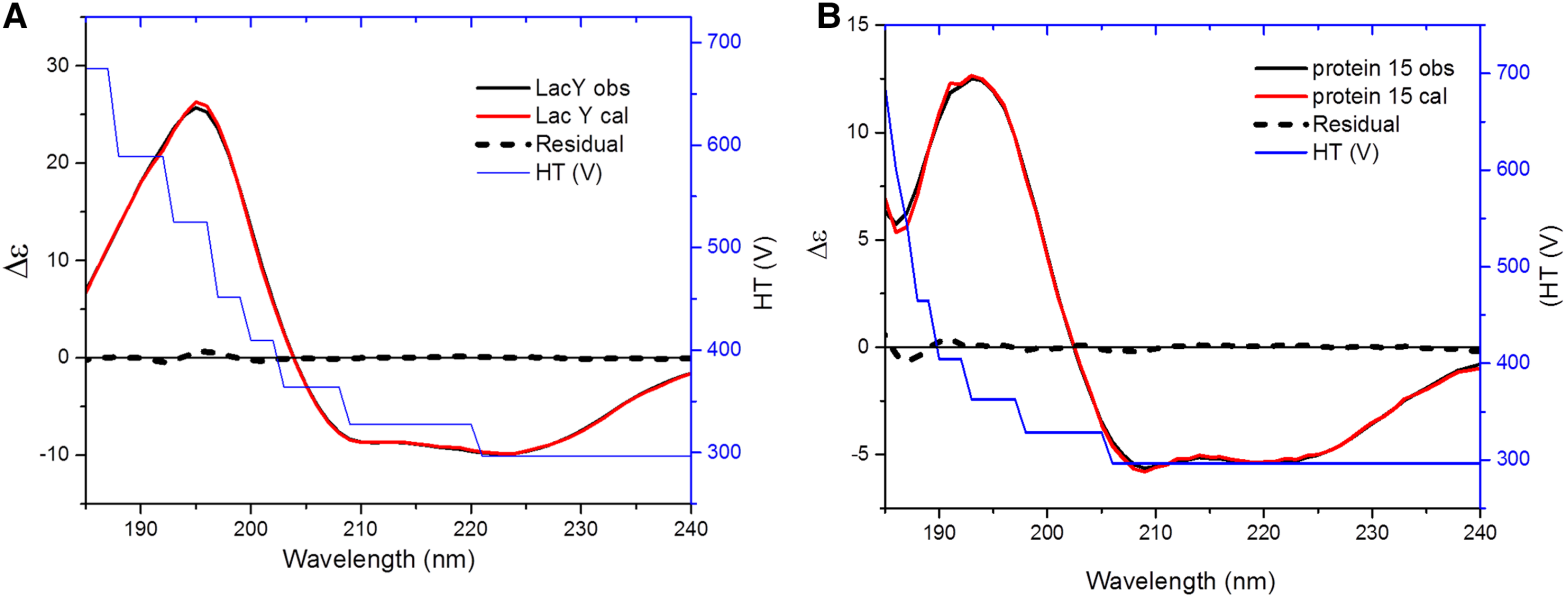
CD spectra of LacY and Protein 15 in high salt concentration. (**A**) LacY 7.34 mg/ml in 300 mM NaCl, Tris 10 mM, 0.05% DDM, 97% α-helix, <1% β-strand, 3% disorder. (**B**) Protein #15 4.4 mg/ml in 10 mM CHES pH 9.5, 500 mM NaCl, MgCl_2_, 1 mM β-mercaptoethanol, 10% glycerol, 0.5% Cymal-5 with protein secondary structure estimated to be 66% α-helix, 5% β-strand, 9% β-turn and 20% disorder.

These guidelines would ensure the CD spectra obtained to a good quality in the far-UV region of 185 nm to enable protein secondary structure estimation with the small nominal mean residual standard deviation (see example Figure 10 below of LacY and another membrane protein coded Protein 15) (Hussain and de Moraes, personal communication).

3) If **reducing agent** is required, **TCEP** (tris(2-carboxyethyl)phosphine) or **β-mercaptoethanol** is recommended. **DTE** (dithioerythritol) or **DTT** (dithiothreitol) should be avoided as DTE and DTT have UV spectral contribution at ∼260–280 nm that changes with time differently when the protein is present [82,83], and hence, the accuracy of protein concentration will be affected if determined spectroscopically.
4) For **near-UV** (250–330 nm) CD measurements, the pathlength commonly used is 1 cm. Here, optimum protein concentration to be used should be the one that gives at 280 nm, a UV absorption of ∼0.8. The amount of protein in this case can be calculated following the von Hippel method [84]. A good approximation to calculate the extinction coefficient of a protein is to add the contribution of each Tyr (1550), each Trp (5500) and number of disulfide bonds (200).
5) For **CD titrations** where the ligand do contribute to the UV absorption at 280 nm, a reduced protein concentration of 0.4 should be used that can be increased by the addition of the ligand up to 1.5. In this case, if the titration is conducted adding small aliquots to an initial volume of protein solution, for example, 0.200 ml, a final volume of the protein–ligand mixture should not exceed 20% of the initial volume reaching maximum a volume of 0.220 ml. Hence, it is recommended for the ligand to be prepared as 10–20× concentration of stock solution. Small aliquots of the ligand can then be added to the protein host, ensuring the final stoichiometric protein : ligand ratio and mixture does not exceed protein dilution of 20%. This is to avoid any conformational or local tertiary structural changes due to concentration effect as opposed to ligand binding.
6) Thorough mixing of the protein : ligand mixture should be carried out gently with careful pipetting to avoid any formation of air bubbles. Incubate the mixture to allow stabilisation of any association due to binding. The **incubation time** can be simply determined by measuring consecutive repeated CD spectra until the spectral features are not changing within the spectral noise. Knowing the time for each measurement, the total time can be easily calculated. Longer incubation times indicate a more unstable system, either due to insufficient mixing after the addition of an aliquot of ligand stock solution that can often be attributed to the presence of buffer components such as viscous additives including glycerol and detergent used in the preparation or stabilisation of membrane proteins. The most unstable membrane protein we measured in terms of length of incubation time required for equilibration after aliquot addition of the ligand stock solution was FsrC (Figure 4) due to its low detergent concentration [50].

## Conclusion

Various other biophysical methods such as AUC, NMR, ITC and fluorescence have been described to further understanding the conformational behaviour of membrane proteins and their ligand-binding property. When used together, these techniques can help in dissecting the molecular mechanism of action paving the way for a more efficient drug discovery programme of membrane protein targets.

This review highlights the important role of CD and SRCD spectroscopy in tackling the characterisation of membrane protein folding behaviour as a function of the environment such as solvent composition, detergents, pH and also ligand-binding interactions. The ability of CD and SRCD spectroscopic techniques to probe and screen very quickly the conformational behaviour of small amount of membrane proteins under different environments should be used to identify the more appropriate and relevant conditions and used subsequently with other techniques to achieve high atomic resolution with NMR, association properties, thermodynamic properties and binding interaction with AUC, fluorescence and ITC.

## Abbreviations

APD: avalanche photodiode
AUC: analytical ultracentrifugation
CD: circular dichroism
CHES: *N*-cyclohexyl-2-aminoethanesulfonic acid
cryo-EM: cryogenic electron microscopy
HT: high tension
HTCD: high-throughput CD
ITC: isothermal titration calorimetry
MCD: magnetic circular dichroism
MES: 2-(*N*-morpholino)ethanesulfonic acid
NMR: nuclear magnetic resonance
PBD: Protein Data Bank
PMT: photomultiplier tube
SRCD: synchrotron radiation circular dichroism
TCSs: two-component systems
